# Gastric perforation caused by secondary systemic amyloidosis

**DOI:** 10.1002/ccr3.4254

**Published:** 2021-05-24

**Authors:** Hiroto Yamamoto, Akihiko Yokota, Noriyuki Suzuki, Mitsuhiro Tachibana, Yutaka Tsutsumi

**Affiliations:** ^1^ Department of General Medicine Shimada Municipal Hospital Shimada Japan; ^2^ Department of Diagnostic Pathology Shimada Municipal Hospital Shimada Japan; ^3^ Department of Gastroenterology Shimada Municipal Hospital Shimada Japan; ^4^ Department of Nephrology Shimada Municipal Hospital Shimada Japan; ^5^ Diagnostic Pathology Clinic Pathos Tsutsumi Inazawa Japan

**Keywords:** amyloid A, endocrinology and metabolic disorders, gastric perforation, gastroenterology and hepatology, IgM co‐deposition, nephrology, systemic amyloidosis

## Abstract

Amyloid A amyloidosis secondary to chronic inflammation involves systemic organs and tissues, including the gastrointestinal tract. In the present case, massive amyloid deposit caused gastric perforation. IgM co‐deposition in the glomeruli was another finding of note.

## Graphical abstract

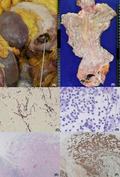

## 1 INTRODUCTION

An aged man, suffering abscess around the replaced femoral prosthesis, complained of intractable watery diarrhea and hematochezia. Autopsy disclosed 12‐mm‐sized perforation at the gastric prepylorus with purulent peritonitis. Amyloid A was deposited in systemic organs and tissues, including the site of gastric perforation. IgM was co‐deposited in the glomeruli.

Amyloidosis is histopathologically characterized by extracellular deposition of water‐insoluble amyloid fibrils that impair the normal organ functions.^1^ In 1853, Rudolf Virchow, the father of modern pathology, first identified systemic deposition of starch‐like material named “amyloid,” which showed staining with iodine and sulfuric acid, dyeing affinity resembling starch.^2^ Amyloidosis is divided into systemic and localized forms, according to the site of involvement. To date, 36 amyloidogenic proteins have been identified.^3^ Systemic amyloidosis encompasses primary AL (immunoglobulin light chain) amyloidosis, amyloid A (AA) amyloidosis, familial (AF) transthyretin amyloidosis, senile transthyretin amyloidosis, cystatin C amyloidosis, and hemodialysis‐related β2‐microglobulin amyloidosis.

In 1971, Benditt and Eriksen^4^ found a novel amyloid protein distinctive from AL amyloid, and termed AA. AA amyloidosis resulted from prolonged chronic inflammation. Serum amyloid A (SAA) protein was then identified in cases of secondary systemic amyloidosis.^5^, ^6^

We report here an aged Japanese male patient with gastric perforation caused by AA amyloidosis. The patient manifested severe watery diarrhea and hematochezia. He had persistent infection in the hip around right femoral prosthesis for 1 year. Autopsy disclosed systemic deposition of amyloid A protein, including the gastrointestinal tract and kidney. Remarkable amyloid deposition in the gastric mucosa through the subserosa might have caused gastric perforation. Co‐deposition of IgM with amyloid A protein in the renal glomerulus was the finding of note. Active production of SAA in stimulated hepatocytes and acinar cells of the pancreas and salivary gland is also discussed.

## 2 CASE REPORT

An 82‐year‐old Japanese man was transferred to the emergency unit of Shimada Municipal Hospital, Shimada, Shizuoka, Japan, with complaints of severe watery diarrhea and hematochezia. The patient had suffered from hypertension and hyperlipidemia since the age of 50. He had undergone total hip replacement for right femoral head fracture caused by a traffic accident at the age of 71, and he received a surgical procedure for lumber spinal stenosis at the age of 74. Borderline diabetes mellitus with HbA1c levels ranging from 6.0% to 6.6% was pointed out since the age of 72, while the blood glucose level remained within a normal range. At the age of 76, the diagnosis of hypertensive and diabetic chronic kidney disease was made. The patient suffered from pneumonia when he was 78 years old. One year before admission, an infected cyst (abscess) around the right femoral prosthesis was indicated by fluorodeoxyglucose‐positron emission tomography. *Staphylococcus aureus* was cultured, and the first and third‐generation Cefem plus new quinolone were continuously administered. Three months before admission, continuous drainage from the infected lesion was conducted. No gastrointestinal complaints were recorded prior to the final admission.

**FIGURE 1:**
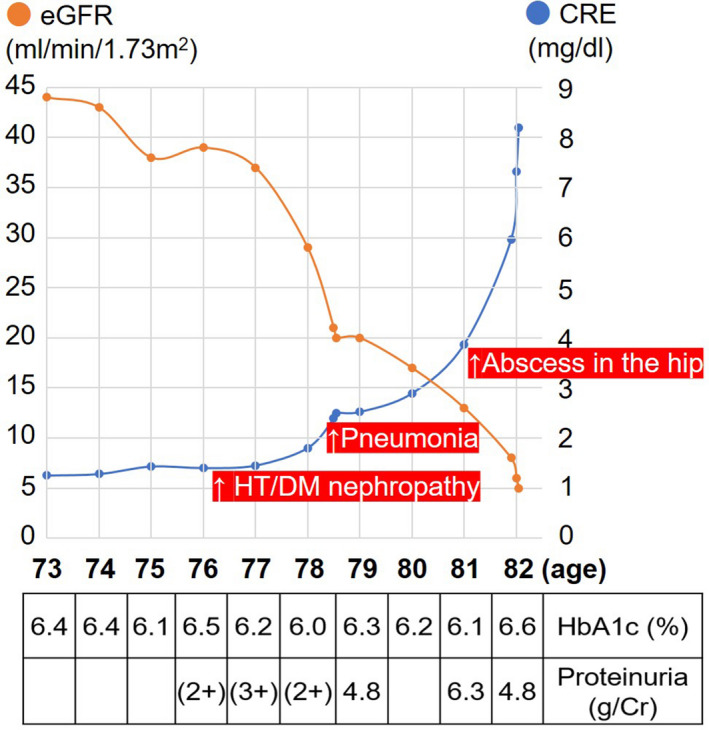
Renal function change transition graph during the last 10‐year period. Values of estimated glomerular filtration rate (eGFR) and serum creatinine (CRE) are plotted, together with hemoglobin A1c (HbA1c) and proteinuria. Reciprocal exacerbation of eGFR and CRE is evident in the latest 5 years. Hemodialysis was performed for the last 3 months. Elevated levels of HbA1c and proteinuria persisted. HT/DM nephropathy: hypertensive/diabetes mellitus‐related nephropathy

His watery diarrhea was severe, more than 10 times a day, and hematochezia was associated. The body temperature was 37.5°C, blood pressure 138/90 mm Hg, heart rate 100 beats per minute, and oxygen saturation 96% while breathing ambient air. The body weight was 57.0 kg with a body mass index at 22.0. Systolic heart murmur was auscultated. The abdomen was soft with weak bowel sounds. There was tenderness of the whole abdomen on palpation, without guarding, rigidity, distention, or mass formation. Main results of laboratory tests on admission included the following: white blood cells 4400/μL, hemoglobin 13.3 g/dL, platelets 305 000/μL, Na 135 mmol/L, K 3.6 mmol/L, Cl 102 mmol/L, Ca 6.4 mg/dL, Pi 4.6 mg/dL, albumin 1.8 g/dL, blood urea nitrogen 49.9 mg/dL, creatinine 5.3 mg/dL, and C‐reactive protein 26.1 mg/dL. The electrocardiogram showed a normal sinus rhythm with long PR and QT intervals. Deterioration of renal functions for the last 10‐year period is illustrated in Figure 1. For the last 3 months, hemodialysis was needed.

**FIGURE 2:**
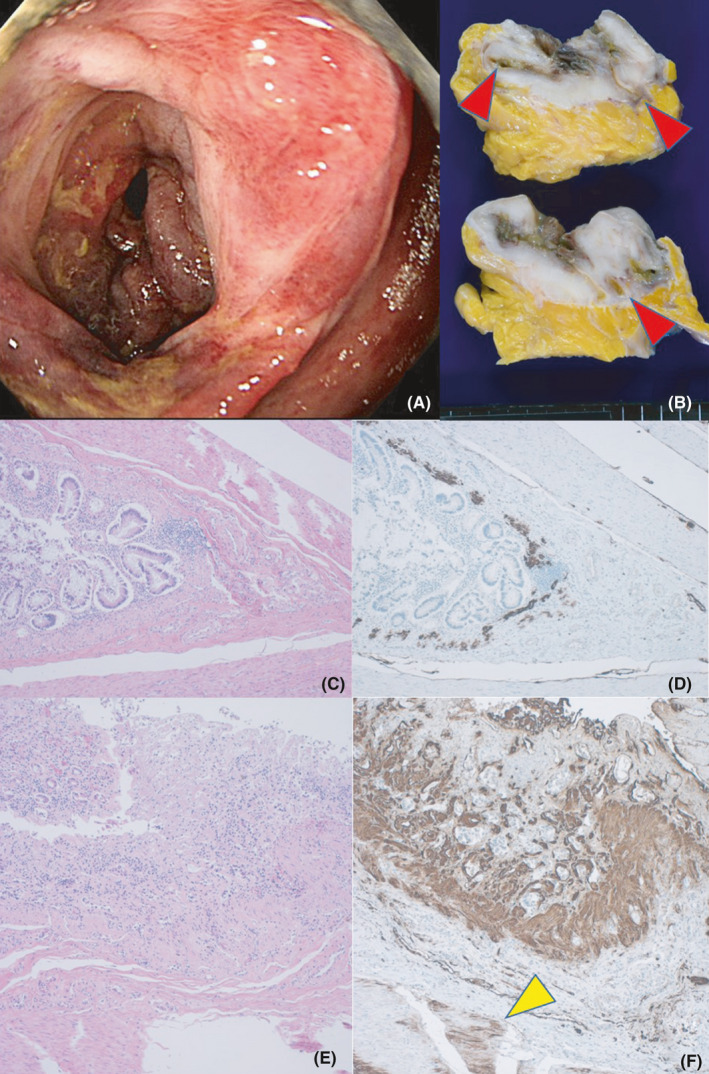
Fibrosing diverticulitis of the sigmoid colon. A, Colonofiberscopic findings, B, gross findings: cut surfaces of the fibrosing sigmoid colon with diverticulosis, C‐F, microscopic features of the diverticulum (C, D) and eroded colonic mucosa (E, F); C and E; H&E, D and F, amyloid A immunostaining. Endoscopically, the sigmoid colon is stenotic with mucosal ischemia and hemorrhagic erosions. Gross autopsy findings indicate that multifocal diverticula (red arrowheads) have provoked mural fibrosis and stenosis. Microscopically, amyloid A deposition is seen along the diverticulum and in the eroded colonic mucosa through the proper muscle layer (yellow arrowhead). The amyloid was massively deposited in the mucosa

Plain and contrast‐enhanced computed tomography scans, performed on the first and eighth days of hospitalization, respectively, indicated significant ischemic changes of the sigmoid colon through the rectum with wall thickening of the sigmoid colon, ascites retention, bilateral pleural effusions, and mild renal atrophy. Colonofiberscopy indicated diverticulosis in the sigmoid colon and ischemic changes and erosions of the stenotic sigmoid colon through rectum (Figure 2A). Microbiologically, the stool proved normal flora, and fecal *Clostridium difficile* toxin test and blood microbial culture were negative. The clinical diagnosis of ischemic and infectious colitis secondary to the long‐term usage of antibiotics was made. Intravenous infusion of ceftriaxone for 5 days was chosen empirically because it is metabolized in the liver. However, it was ineffective for relieving diarrhea and colitis, and then meropenem was chosen for controlling the intractable colonic symptoms. Inflammation peaked out 14 days after the treatment, but soon, the inflammatory reaction and diarrhea re‐exacerbated. The patient expired 26 days after admission. No free air was observed in the abdominal cavity on the contrast‐enhanced computed tomography scan 3 days before death.

**FIGURE 3:**
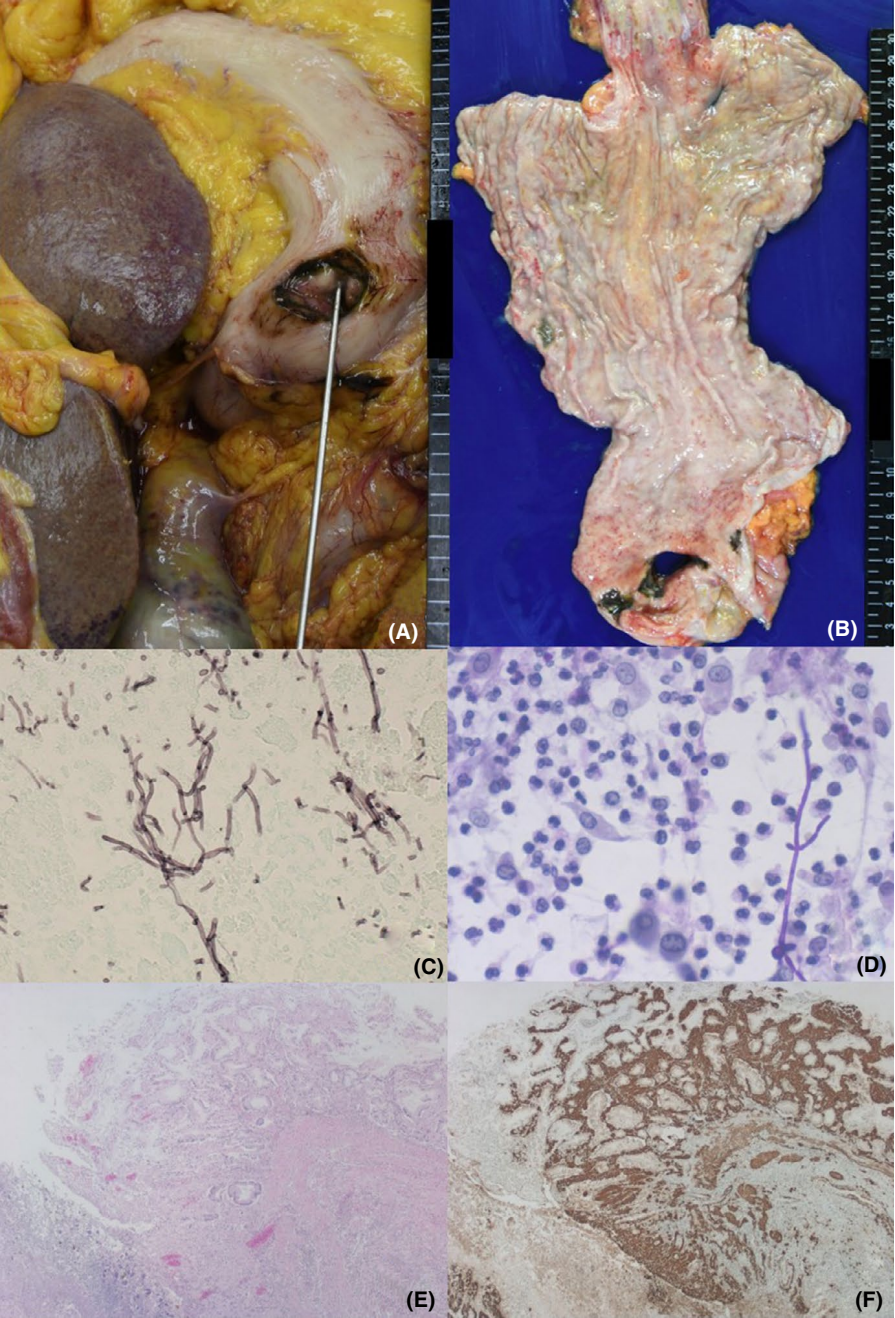
Gastric perforation with Candida infection and massive amyloid deposition. A, B, Gross findings at autopsy (the view from the serosal side [A] and from the mucosal side [B]), C, Candida infection on the serosa (Grocott), D, Candida growth in ascitic fluid (Giemsa), E, F, Amyloid A deposition in the gastric mucosa at the site of perforation (E, H&E, F, amyloid A immunostaining). A 12 mm‐sized perforated hole is grossly seen at the prepylorus of the stomach. A probe is inserted through the gastric perforation at the in situ position. Dissemination of Candida on the peritoneum is proven with Grocott and Giemsa staining. Amyloid deposition is especially prominent in the gastric wall at the site of perforation

The autopsy was conducted 6 hours after death. In the sigmoid colon, fibrosing and adhesive diverticulitis with mucosal ischemic changes and erosions were grossly observed. Microscopically, eosinophilic hyaline material was deposited throughout the gut wall. The deposits were congophilic. Immunohistochemically, amyloid A, visualized by the monoclonal antibody mc‐1 against serum amyloid A1 (SAA1),^7^ was identified in the lamina propria mucosae through the subserosa, including diverticular and vascular walls (Figure 2B–F). In the prepylorus of the stomach, a large perforating ulcer measuring 12 mm was found, and purulent peritonitis with turbid ascites was associated (Figure 3A&B). Candida infection was microscopically demonstrated on the involved mucosa and serosa at the site of gastric perforation (Figure 3C&D). *Candida* *albicans* were cultured from turbid ascitic fluid containing a total of 1150 mL. Candidal esophagitis was also noted. Deposition of amyloid A was evident in the gastric mucosa through the subserosa, and the amyloid deposition was especially prominent and seen in the full‐thickness of the mucosa at the site of gastric perforation (Figure 3E&F). Amyloid A was also diffusely deposited in the wall of the esophagus through the rectum. Pleural effusion was associated (left: 150 mL, right: 300 mL).

Systemic congophilic deposition of amyloid A was further demonstrated in the tongue, salivary gland, thyroid gland, lung, heart, pancreas, splenic white pulp, adrenal glands, kidney, urinary bladder, and ureter. The deposition was especially pronounced in the thyroid gland (38 g) and urinary tract.

**FIGURE 4:**
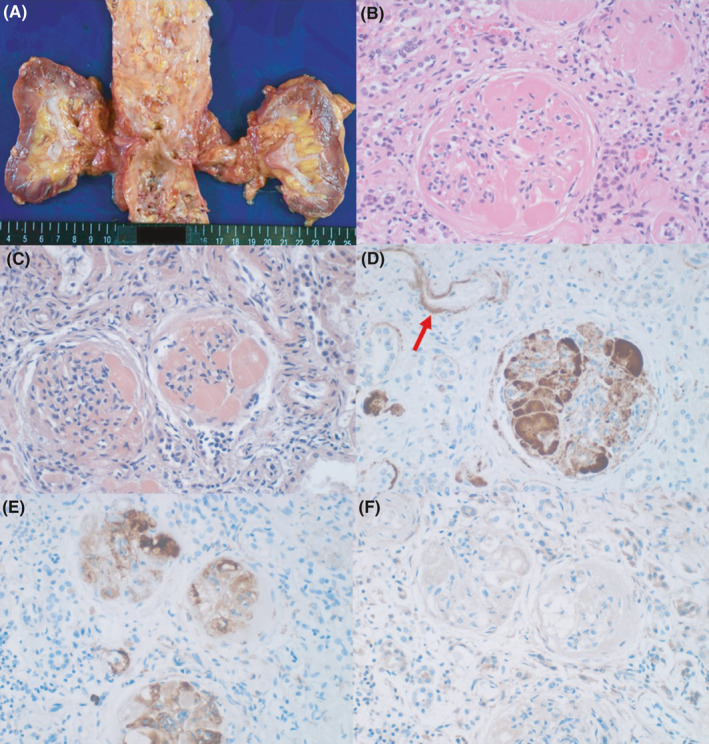
Renal amyloidosis. A, Gross appearance, B, H&E, C, Congo red, D‐F, immunostaining for amyloid A (D), IgM (E), and IgG (F). Both kidneys are atrophic with thinning of the renal cortex. Aortic atherosclerosis is severe in degree. The glomerular eosinophilic deposits are congophilic and immunoreactive for amyloid A. Amyloid deposition is also noted in the vascular wall (arrow). The intraglomerular deposits are also stained for IgM, but not for IgG. Immunostaining using formalin‐fixed, paraffin‐embedded sections after prolonged protease‐1 digestion clarified co‐deposition of amyloid A and IgM

**FIGURE 5:**
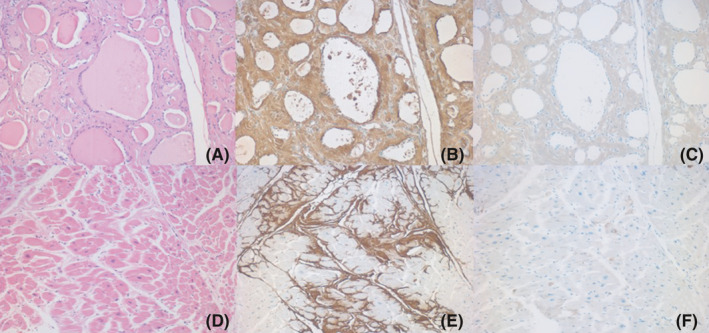
Amyloid deposits in the thyroid (A‐C) and heart (left ventricle: D‐F). A and D: H&E, B and E: amyloid A, C, and F: IgM after prolonged protease‐1 digestion. The stromal amyloid deposit in the thyroid and heart is strongly positive for amyloid A, but co‐deposition of IgM is not observed in the extra‐renal sites

Atrophic kidneys weighed 72 g (left) and 73 g (right). Microscopically, amyloid A deposits caused amyloid glomerulopathy in association with vascular wall involvement. Features of diabetic glomerulosclerosis were indistinct because of marked amyloid deposition, while arteriolosclerosis was observed. Pancreatic islets focally showed diabetes‐related localized hyaline (amyloid) deposition. Characteristically, co‐deposition of amyloid A and IgM in the renal glomerulus was demonstrated by immunostaining using formalin‐fixed, paraffin‐embedded sections after prolonged protease‐1 digestion ^8^ (Figure 4). IgG and IgA were undetectable. The co‐deposition of amyloid A and IgM was scarcely observed in the systemic amyloid A deposits outside the kidney (Figure 5).

**FIGURE 6:**
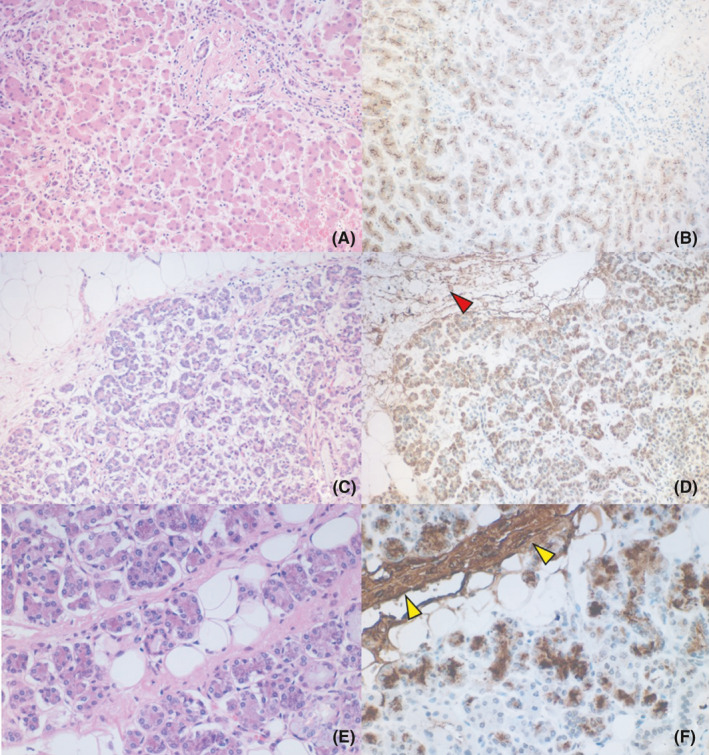
Accelerated production of serum amyloid A1 (SAA1) by the stimulated hepatocytes (A, B), pancreatic acinar cells (C, D), and submandibular gland acinar cells (E, F). A, C, and E: H&E, B, D, and F: immunostaining for amyloid A (SAA1). The cytoplasm of the hepatocytes and acinar cells of the pancreas and salivary gland is granularly decorated brown with the monoclonal antibody mc‐1 against SAA1. Amyloid A was deposited in the peripancreatic adipose tissue (D, red arrowhead) and in the salivary gland stroma (F, yellow arrowheads)

Most of the hepatocytes were immunostained with the anti‐SAA1 monoclonal antibody mc‐1. Cytoplasmic granular positivity was evident. The acinar cells of the pancreas and salivary gland were also positively stained. These findings indicated accelerated production of SAA1 protein by these cells as a prolonged inflammatory response (Figure 6).

Another incidental finding included aortic stenosis caused by dystrophic calcification in the cusps of the aortic valve. Left ventricular hypertrophy (heart weight 415 g) was caused by both aortic stenosis and amyloid deposition. The aorta revealed severe atherosclerosis. The abscess lesion around the right femoral prosthesis was not evaluated.

## 3 DISCUSSION

We described herein a case of gastric perforation secondary to systemic AA amyloidosis. Persistent infection around the right femoral prosthesis for 1‐year period was regarded as the amyloidogenic chronic inflammatory event. Continuous drainage from the abscess lesion was performed for the last 4 months. Chronic fibrosing diverticulitis in the sigmoid colon was another candidate of the amyloidogenic inflammatory lesion in the present case.

Okuda, et al^9^ reported that the most frequent underlying inflammatory disease provoking AA amyloidosis was rheumatoid arthritis (60.3%). Chronic infection, including postoperative refractory infection, caused AA amyloidosis in 4.5% of cases.^10^ Li, et al^11^ described a case of chronic diverticulitis inducing AA amyloidosis. In the past, tuberculosis and leprosy were the major source of AA amyloidosis.^12^, ^13^ It has been reported that people over 70 years of age are susceptible for developing AA amyloidosis after chronic inflammation lasting for a relatively short period of time (just a few years).^14^

It is of note that hepatocytes and acinar cells of the pancreas and salivary gland showed granular cytoplasmic immunoreactivity of SAA1. The findings may reflect accelerated secretion of SAA1 in response to prolonged chronic inflammation. Reportedly, SAA1 production in the hepatocyte is increased more than 1000 times after persistent inflammatory stimulation, when compared with the normal (unstimulated) status.^15^ Urieli‐Shoval, et al^16^ illustrated widespread SAA1 messenger RNA (mRNA) expression in a variety of normal epithelial cells by in situ hybridization technique. Recently, attention has been focused on the production of SAA1 by neoplastic cells. A representative one is inflammatory hepatocellular adenoma, in which SAA1 functions as a diagnostic immunohistochemical marker.^17^ Varied types of cancer cells also produce and secrete SAA1.^18^, ^19^, ^20^

Serum amyloid A, consisting of four different isoforms (SAA1‐4), belongs to the family of apolipoprotein in high‐density lipoprotein (HDL).^21^, ^22^ SAA plays an important role in HDL metabolism and cholesterol homeostasis. SAA1 and SAA2 are categorized in the acute phase protein synthesized in the hepatocyte in response to inflammation. SAA3 is thought to be encoded by a pseudogene, and SAA4 is constitutively expressed in the hepatocyte as a component of HDL. SAA1 is the major constituent of amyloid A protein, consisting of the N‐terminal segment of SAA1 of variable length. Interleukin‐6 (IL6) is the most powerful inflammatory cytokine activating the expression of SAA1 mRNA in the hepatocyte. IL1 and tumor necrosis factor‐alpha reveal a synergistic effect on the mRNA expression induced by IL6.^23^

A subset of (not all) patients with persistent chronic inflammatory disease develop AA amyloidosis. The SAA1 gene encodes five polymorphic alleles (SAA1.1‐SAA1.5), producing proteins with minor amino acid substitutions.^21^ Such SAA1 polymorphism is considered to have an effect on the amyloidogenesis. In Japanese patients, the SAA1.3 allele represents a high‐risk factor of AA amyloidosis.^24^ In Caucasian patients with rheumatoid arthritis, SAA1.1 facilitates developing AA amyloidosis.^25^

The most frequent clinical manifestations of AA amyloidosis are related to renal dysfunction with nephrotic syndrome and gastrointestinal involvement such as intractable diarrhea and hematochezia. Gastrointestinal amyloidosis provokes erosion, ulceration, bleeding, perforation, pneumatosis intestinalis, malabsorption, and paralytic ileus (dysmotility).^26^, ^27^, ^28^ In the present case, watery diarrhea and hematochezia were caused by massive deposition of amyloid A protein in the gastrointestinal tract.

The direct cause of death of the present case was amyloid deposition‐related gastric perforation. Gastrointestinal perforation is a rare occasion in systemic amyloidosis.^29^, ^30^ Ganzoni and Schneider^31^ described a case of gastric perforation due to primary amyloidosis in 1981. To the best of our knowledge, this is the second case of gastric perforation caused by systemic amyloidosis. Remarkable deposition of amyloid A protein in the prepyloric mucosa through the subserosa might have provoked gastric perforation.

It should be noted that in the glomerular lesion of the atrophic kidney, IgM was consistently co‐localized with the amyloid A deposit. The co‐deposition was not seen in the extra‐renal amyloid lesions. Ayar, et al^32^ reported that the glomerular deposition of IgM on amyloid A was not correlated with the patient's outcome in AA amyloidosis. Glomerular IgM deposition has been observed in glomerulosclerosis of the secondary form, including diabetic nephropathy,^33^ hypertensive nephropathy,^34^ and focal segmental glomerulosclerosis.^35^ In an animal model of glomerulosclerosis, IgM activates the complement system in the glomerulus.^35^ In another experimental model of a nonsclerotic and nonimmune complex glomerular disease in mice deficient for the complement regulatory protein factor H, IgM was bound to neo‐epitopes on the insulted glomerulus and exacerbated the disease.^36^ Further studies are needed to clarify the pathophysiological significance of co‐deposition of IgM in AA amyloid glomerulopathy.

## CONFLICT OF INTEREST

None declared.

## AUTHOR CONTRIBUTIONS

We declare that all the authors 1) made a substantial contribution to the concept of the case report or interpretation of data, and 2) approved the version to be submitted. 3) Each author has participated sufficiently in the work to take public responsibility for appropriate portions of the content. HY, MT, and YT: analyzed the autopsy findings. AY and NS: contributed to clinical observation and care.

## ETHICAL APPROVAL

All the procedures were in accordance with the ethical standards of the responsible institutional committee on human experimentation and with the Helsinki Declaration of 1964 and later versions. The patient's wife gave a written informed consent to publication of the case report.

## Data Availability

The datasets generated during and/or analyzed during the current study are available from the corresponding author on reasonable request.
